# Trigeminal trophic syndrome: Strange evolution of maxillofacial surgery

**DOI:** 10.17179/excli2019-1846

**Published:** 2019-10-16

**Authors:** Francesca Zotti, Giorgia Capocasale, Fabio Lonardi, Tiziano Zambotti, Riccardo Nocini, Massimo Albanese

**Affiliations:** 1 Department of Surgical Sciences, Paediatrics and Gynecology, University of Verona, Policlinico G. B. Rossi. Piazzale L. Scuro n.10, 37134. Verona, Italy; 2 Department of Otorhinolaryngology - Head and Neck Surgery, University Hospital of Verona, Italy, Policlinico G. B. Rossi. Piazzale L. Scuro n.10, 37134. Verona, Italy

**Keywords:** skin ulcer, trigeminal nerve injury, allergy, osteolysis

## Abstract

Trigeminal trophic syndrome (TTS) is a rare facial/cranial affection that arises in ulcerations, itch and paresthesia. Etiology is debated, however trigeminal nerve damage seems to be frequent in pathogenetic patterns. The disease may affect any region innervated by the trigeminal nerve, especially the maxillary branch. A case of TTS, trigged by allergic reaction to osteosynthetic materials and involving infraorbital nerve, was presented. The feature that makes this case one-off in the literature is the association with osteolytic lesion surrounding infraorbital nerve. Diagnosis and treatment were difficult and multidisciplinary approach was required. Treatments administered were satisfying and signs and symptoms remitted, however patient quitted follow-up. TTS is a rare disease, diagnosis is difficult to be performed and it is often a diagnosis of exclusion. Treatment is challenging and it requires a multidisciplinary approach and a great compliance of patients.

## Introduction

Trigeminal Trophic Syndrome (TTS) is a rare condition caused by peripheral or central trigeminal damages. Although initially described by Wallenberg in 1895 (Wallenberg, 1895[17]), the first cases of TTS were reported in different publications in 1933 (Loveman, 1933[9]; McKenzie, 1933[10]). 

Typical symptoms include unilateral anesthesia/dysesthesia and/or itch in the dermatome/s innervated by the damaged trigeminal branch/es, especially the II.

Typical signs of TTS are unilateral non-healing ulcers on the face, especially located in ala nasi with cartilage erosion.

The most reported recognized causes are outcomes of neurosurgery and cerebrovascular diseases (Ferrara et al., 2001[5]). Iatrogenic causes are also reported: section/Percutaneous Ethanol Injection (PEI) of the Gasserian Ganglion to treat trigeminal neuralgia are associated with TTS (Sadeghi et al., 2004[13]). Moreover, damage from neurotrophic viruses, such as HHV, can lead to TTS (Shea et al., 1996[15]). Whereas post-ganglionic lesions are described as cause of TTS, there are few cases reported in literature.

Pathogenesis of ulcers is debated. Some authors reported self-infliction as main cause of ulcers, because of itch due to anesthesia and paresthesia of the affected side (Rashid and Khachemoune, 2007[12]). However, other authors assumed different pathogenesis of ulcerations, considering that occlusive dressing to prevent scratching is not always effective (Datta et al., 2000[4]; Khan et al., 2017[7]).

Treatment is contested and difficult to set, it includes physical restraint measures and/or drug intake such as gabapentin and carbamazepine (Kabashima et al., 2010[6]).

Table 1(Tab. 1) shows main characteristics of trigeminal trophic syndrome, according to literature. 

In this paper is exposed a case of TTS caused by peripheral nerve damage after an osteolytic lesion surrounding infraorbital nerve, result of a Vitallium® microplate in an allergic patient treated by Le Fort I osteotomy for maxillary hypoplasia. 

## Case Report

A 32-year old woman has been referred to Department of Maxillofacial Surgery of University of Verona (Italy) for recurrent, painful, perioral and intraoral lesions in 2016. 

Clinical history reported a Le Fort I surgery procedure performed to correct Class III dentoskeletal malocclusion in another hospital in 2007. Osteosynthesis of upper maxilla was achieved by means of two Vitallium® microplates. Symptoms and signs occurred 9 years after orthognathic surgery. 

**Figure 1:**
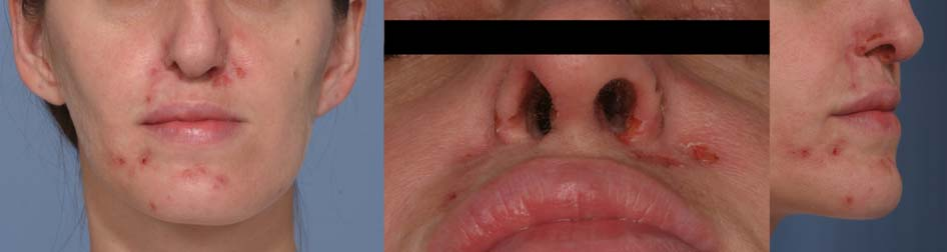
First visit: nasal and paranasal ulcers are evident

Ulcers localized in nasal region, with remarkable damage of ala nasi were detected at first visit (Figure 1(Fig. 1)).

Bacteriological sample and blood tests were normal. Patch tests have been carried out according to recommendations of the International Contact Dermatitis Research Group (ICDRG) with Haye's chambers Test. Readings were evaluated 2 and 3 days later and reactions were scored according to the criteria of the ICDRG. Patient was tested with the European standard series as well as dental materials and a metal series. Only Nickel triggered a positive reaction (strong reaction). Other allergens tested were negative (no reaction).

Since there were no clinical improvements after antibiotic therapy (Amoxicillin and Clavulanic Acid 1gr per 3 times per day for 10 days) and given the possibility of allergy to osteosynthetic materials, surgery was carried out to remove plates.

Intraoperative examination clearly revealed an osteolytic area surrounding infraorbital foramen and involving the infraorbital nerve.

The patient reported an immediate subjective relief after surgery; itch and pain were reduced, although paresthesia was still present at hospital discharge. 

Intraoral and perioral lesions progressively healed and they were absent 6 months after surgery, however paresthesia of infraorbital nerve did never disappear. 

Given the intraoperative finding of osteolytic area surrounding the nerve, functional damage of trigeminal nerve was tested by a neurological examination (clinical examination and cerebral potential tests) in a dedicate check, all neurological tests performed pointed out damage of II and III branches of the trigeminal nerve and an increased perceptive threshold, whereas blink reflex was normal. 

The neurologist suggested a clinical follow-up to evaluate evolution of paresthesia, however, due to poor compliance of our patient, this follow-up has never been finalized.

One year after microplates removal, ulcers on the lower-right part of the face and alteration of the nasal profile were detected again. The patient also reported pain in these areas. A further evaluation by an anesthetist specialized in pain management was carried out, however the patient refused to take drugs and to be involved in a follow-up program.

**Figure 2:**
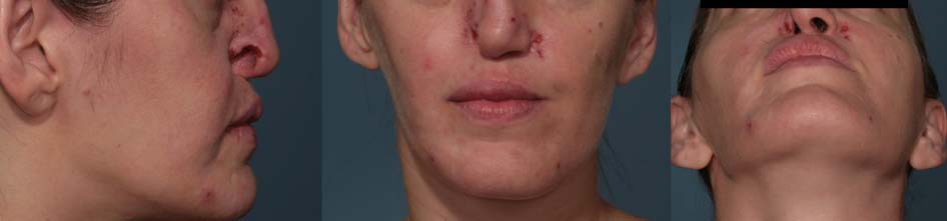
Worsening of the nasal and skin ulcers

Four years later patient asked one more our department for a new re-evaluation, due to the worsening of the skin face lesions (Figure 2(Fig. 2)).

Diagnosis of TTS was made by a multidisciplinary approach involving a dermatologist, oncologist, maxillofacial surgeon and an anesthetist. The young woman was treated in sequence for allergic dermatitis/stomatitis and trigeminal trophic syndrome. A therapy for TTS based on tacrolimus (off-label) to stabilize skin lesions and gabapentin to reduce itch (Kabashima et al., 2010[6]) was administered. A further evaluation was scheduled 3 months after the beginning of drug therapy and a great improvement in symptoms was highlighted, confirming our diagnosis ex-adiuvantibus.

Six months later, a surgery to reduce the nasal defect was proposed to patient (Bertossi et al., 2007[2]). She initially accepted the surgery, however 2 months later she refused and quitted the follow-up.

## Discussion

Microplates and screws are often used for rigid fixation in orthognathic surgery. 

The most commonly used hardwires contain titanium, however microplates made of Vitallium® were frequently used about fifteen years ago. Allergic reactions to this metal alloy have been reported in orthodontic and prosthetic patients (Saglam et al., 2004[14]; Counts et al., 2002[3]). Nevertheless, allergy caused by plates and screws used in Le Fort I osteotomy is of rare occurrence. It is proven that allergy to Nickel can be associated with allergies to other metals, mostly chromium and cobalt (Uter et al., 2016[16]; Lidén et al., 2016[8]). Plates and screws used in the fixation of the upper maxilla were made of Vitallium® (65 % Cobalt, 30 % Chromium, 5 % Molybdenum). 

Vitallium® appliances release continuously particles into surrounding tissues leading to a delayed inflammation, which may appear 4 to 6 years after surgery. 

Skin signs of allergies to metal range from rashes to inflammation with mild erythema to a fiery red color, with or without edema are present. Usually symptoms and signs appear six/seven years after surgery and disappear after removal of metal plates (Lidén et al., 2016[8]). 

In the case we reported, intraoral and perioral lesions appeared nine years after Le Fort surgery, probably due to spreading of metal particles trough soft and hard tissues (Park and Shearer, 1983[11]; Bertoldi et al., 2005[1]). Patients who are suspected of having metal allergies should be referred for patch testing. 

In the case we reported, the continuous inflammation caused by the allergy to the metals in microplates resulted in an area of osteolysis around the infraorbital nerve, which produced damage to the nerve itself. Peripheral trigeminal damage started with an area of dysesthesia in the lower-right region of the patient's face, afterwards, probably due to the persistence of these symptoms, it developed into TTS. 

The most used therapies reported in literature are occlusive bandage alone or occlusive bandage associated to pharmacological treatment (Ferrara et al., 2001[5]; Sadeghi et al., 2004[13]; Shea et al., 1996[15]; Kabashima et al., 2010[6]).

## Conclusions

TTS is a rare and difficult condition to identify and treat. Other treatable diseases must be excluded in order to reach the diagnosis of TTS. Diagnostic procedures should include biopsies, laboratory tests and imaging. Treatment options are different and a multidisciplinary approach is required. Further studies are needed to identify pathogenesis and the best way to treat this disease.

## Disclaimer

The patient involved in this study gave informed consent for utilization of her personal data.

## Conflict of interest

The authors have no conflict of interest to disclose.

## Figures and Tables

**Table 1:**
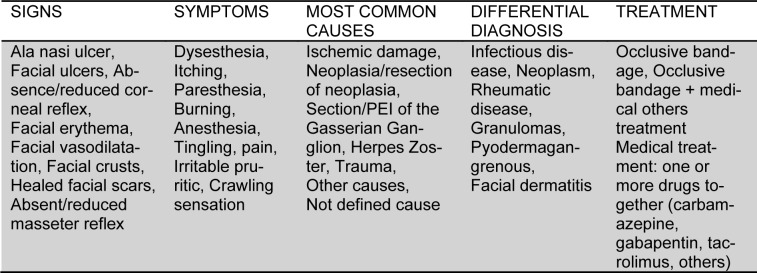
Overview of characteristics of TTS
